# Rethinking Reconstruction: Ethical Standards and Practice Guidelines as a Prerequisite to Clitoral Reconstruction Following Female Genital Mutilation/Cutting

**DOI:** 10.1093/asj/sjab383

**Published:** 2021-11-02

**Authors:** Jasmine Abdulcadir, Emily Manin, Brian D Earp, Elizabeth M N Ferguson, Dan mon O’Dey, Crista E Johnson-Agbakwu

**Affiliations:** 1 Department of Pediatrics, Gynecology and Obstetrics, Geneva University Hospitals, Geneva, Switzerland; 2 Weill Cornell Medical College, New York, NY, USA; 3 Yale-Hastings Program in Ethics and Health Policy, New Haven, CT, USA; 4 Department of Plastic and Reconstructive Surgery, Valleywise Health Medical Center, Phoenix, AZ, USA; 5 Department of Plastic Surgery, Luisenhospital Aachen, Aachen, Germany; 6 Refugee Women's Health Clinic, Valleywise Health, Phoenix, AZ, USA

Wilson and Zaki describe a “Novel Clitoral Reconstruction and Coverage With Sensate Labial Flaps” as a “potential remedy” for women who have undergone female genital mutilation/cutting (FGM/C).^1^ We would like to discuss some scientific and ethical issues in relation to clitoral reconstruction (CR) surgery, touching on sociocultural, political, interpersonal, and psychological factors involved in promoting psychosexual health of women and girls with FGM/C.

## EVOLUTION OF CLITORAL RECONSTRUCTION TECHNIQUES

Since the first publications on CR, several modifications, including microsurgical approaches, have been proposed to the original technique (Table 1), but few rigorous studies have been performed to evaluate them.^2,3^ Wilson and Zaki propose a modification of the Foldès technique with no sectioning of the suspensory ligament and with a “sensate labial flap” from the uncut or less-cut labium. However, trade-offs must be considered: the proposed technique involves (further) cutting of healthy labial tissues, introducing surgical risk to another erogenous area of the vulva. Additionally, the implications of the interactions between the clitoral and transposed labial tissue are unclear. The study also does not demonstrate that the technique achieves reproducible results concerning both form and function. Although we acknowledge the existing diversity of shapes and sizes of vulvas and clitorises, the pictures presented in the paper show “reconstructed” clitorises that seem enlarged compared with what is characteristic for healthy vulvar anatomy without FGM/C.^4^ However, the authors do not report measurements of the re-exposed clitoral body in their patients and do not mention their patients’ preferences for a specific size for their re-exposed clitoral body, as has been described in previous literature.^5,6^

**Table 1.** Existing Clitoral Reconstruction Techniques

Authors	Specialty	Technique (summary)
Thabet (Egypt) 2003, Foldès (France) 2004	Gynecologists, urologist	Removal of the cutaneous scar tissue covering the clitoral stump and subsequent dissection and mobilization of the clitoral body through the section of the suspensory ligament of the clitoris. The body of the clitoris is then anchored to the bulbocavernosus muscles in a lower, accessible, and visible position. The re-exposed clitoris will be re-epithelialized in approximately 3 months.
Ouedraogo (Burkina Faso) 2016	Gynecologist	Modified technique of Thabet and Foldès that does not involve the suture of anchoring the re-exposed clitoris to the bulbocavernous muscles.
O’Dey (Germany) 2017	Plastic surgeon	More complex technique that includes an anterior obturator artery perforator flap (aOAP-flap) for vulvar reconstruction, an omega domed flap (OD flap) as surgical access to the clitoral organ and for clitoral foreskin reconstruction, and a microsurgical procedure called neurotization and molding of the clitoral stump (NMCS procedure) that frees, transposes, and fixes the nerve branches of the dorsal nerve of the clitoris at the end of the re-exposed clitoral body (renewed clitoral tip).
Chang et al (USA) 2017	Plastic surgeons	Wide circumferential dissection of the superficial scar between the labia followed by deep dissection to free the clitoris from the pubic bone. The labia majora are then rolled up and sutured to the periosteum. The clitoris becomes re-epithelialized over time, and a non-adhesive dressing is applied to prevent the clitoris from adhering to the surrounding tissues. There is no section of the suspensory ligament of the clitoris.
Mañero and Labanca (Spain) 2018	Plastic surgeons	Like the Foldès technique, it involves the removal of scar tissue, the section of the suspensory ligament of the clitoris and the anchoring of the clitoris in a lower position. However, the re-exposed clitoris is covered with a mucosal graft from the posterior vaginal wall.
Wilson et al (Egypt) 2021	Plastic surgeons	Modified technique of Thabet and Foldès without section of the suspensory ligament of the clitoris and with covering of the re-exposed clitoral body with a labial flap obtained from one of the inner labium.
Botter et al (France) 2021	Plastic surgeons	Modified technique of Thabet and Foldès without resection of the skin scar above the clitoris but with reverse V incision and microsurgical dissection of the clitoral body.

## ASSESSMENT OF FEMALE SEXUAL RESPONSE

We have concerns about the seemingly subjective nature of some of the claims made in Wilson and Zaki’s article. To assess female sexual function and experience in the patient sample, the authors employ a Scale for Clitoral Sensation, a non-validated questionnaire from Foldès’s first paper that has been criticized by experts since.^3,5,7^ The authors also deem the postoperative clitoris to be “good-sized” without employing a validated measure. Wilson and Zaki assert that patients felt “more confident,” “were glad they had the surgery,” and had “better self-esteem” after CR without systematically evaluating and assessing patient experiences.

More generally, the authors seem to adopt a clitorocentric lens in conceiving of female sexuality. As noted, the surgery may compromise sensation in the labia in pursuit of clitoral surgery and enhancement of pleasure. The study could have acknowledged the complex multi-dimensionality of female sexual pleasure and satisfaction, which encompasses biochemical, anatomic, neurophysiologic, cognitive, interpersonal, and sociocultural influences.^8^

## IATROGENIC PATHOLOGIZATION

We are concerned with some of the language utilized to describe the women with FGM/C in the target paper. For instance, the paper describes individuals with FGM/C as “poor victims,” which might be seen as condescending. Although many women do feel victimized by the practice of FGM/C, others do not and regard such language as stigmatizing.^9,10^ Recent evidence links such stigmatization to decreased well-being, sense of identity, self-esteem, and genital self-image among women with FGM/C living in low-prevalence diaspora countries.^11^ Indeed, clitoral surgery is often requested as a repair of stigma.^12^ When health care providers employ terminology that assumes or projects victim status on women seeking care, this may negatively impact patient experience and could compromise the decision-making process surrounding clitoral reconstruction. Recent research points to a complex interplay between women’s attitudes, perceived social norms, and a sense of control over one’s own narrative, all of which bear on decisions surrounding clitoral reconstruction, as captured the Theory of Planned Behavior by Brady et al.^8^ When women make an appropriately informed decision to undergo clitoral reconstruction, they start a journey of self-reconstruction, which is only partially surgical.

With this complex psychosocial landscape in mind, we agree with the authors’ sentiment that multidisciplinary teams with expertise in caring for women with FGM/C, particularly those undergoing clitoral reconstruction, are necessary. These multidisciplinary teams should include individuals with expertise in the sociocultural, political, and anthropological implications of FGM/C.

## CONCLUSIONS

Moving forward, we propose that ethical standards for research and care of women with FGM/C be enacted.^13^ Multidisciplinary experts including women with FGM/C should establish recommendations that delineate culturally and linguistically appropriate, non-stigmatizing counseling, education, and shared decision making.^14^ Algorithms should be created with clear criteria for surgical interventions, and the utilization of validated instruments for assessing sexual function, clinical outcomes, genital self-image, and patient satisfaction should become standard. The creation of patient registries will allow long-term safety and efficacy data to be tracked across distinct surgical techniques, establishing consistent quality metrics. The first meeting of surgeons who perform clitoral reconstructive surgery in Europe, organized by the European network *End FMG*, is to be held in November 2021 and is the first step towards a standard of care.
